# Supplementary material for: Multiple inputs ensure yeast cell size homeostasis during cell cycle progression
Source: eLife. 2018 Jul 4;7:e34025. doi: 10.7554/eLife.34025 (PMC6085122; doi:10.7554/eLife.34025)
Supplement: Supplementary file 1. [file elife-34025-supp1.docx]

**Strain list**

| Name | *Background* | *Relevant Genotype* | *Genotype* | Origin |
| --- | --- | --- | --- | --- |
| YCG01 | *S288C (BY4741)* | *HTB2-sfGFP* | *BY4741; MATa; ADE2; his3Δ1; leu2Δ0; lys2Δ0; met15Δ0; ura3Δ0; TRP1; HTB2-sfGFP::kanMX4* | This study |
| YCG02 | *S288C (BY4742)* | *HTB2-sfGFP* | *BY4742; MATα; ADE2; his3Δ1; leu2Δ0; lys2Δ0; MET15; ura3Δ0; TRP1; HTB2-sfGFP::kanMX4* | This study |
| YCG07 | *S288C (BY4742)* | *HTB2-GFP; CDC10-mCherry* | *BY4742; MATα; ADE2; HIS3; leu2Δ0; ura3Δ0;  HTB2GFP::HIS3; CDC10-mCherry::KanMX4* | This study |
| YCG15 | *S288C (BY4741)* | *HTB2-sfGFP; CDC10-mCherry* | *BY4741; MATa; ADE2; his3Δ1; leu2Δ0; LYS2; ura3Δ0; TRP1; HTB2-sfGFP::kanMX4; CDC10-mCherry::KanMX4* | This study |
| YCG16 | *S288C (BY4741)* | *HTB2-sfGFP; CDC10-mCherry* | *BY4741; MATa; ADE2; his3Δ1; leu2Δ0; lys2Δ0; ura3Δ0; TRP1; HTB2-sfGFP::kanMX4; CDC10-mCherry::KanMX4* | This study |
| YCG17 | *S288C (BY4741)* | *HTB2-sfGFP; CDC10-mCherry* | *BY4741; MATα; ADE2; his3Δ1; leu2Δ0; LYS2; ura3Δ0; TRP1; HTB2-sfGFP::kanMX4; CDC10-mCherry::KanMX4* | This study |
| YCG18 | *S288C (BY4741)* | *HTB2-sfGFP; CDC10-mCherry* | *BY4741; MATa; ADE2; his3Δ1; leu2Δ0; LYS2; ura3Δ0; TRP1; HTB2-sfGFP::kanMX4; CDC10-mCherry::KanMX4* | This study |
| YCG19 | *S288C (BY4741)* | *HTB2-sfGFP; CDC10-mCherry* | *BY4741; MATα; ADE2; his3Δ1; leu2Δ0; lys2Δ0; ura3Δ0; TRP1; HTB2-sfGFP::kanMX4; CDC10-mCherry::KanMX4* | This study |
| YCG32 | *W303* | *HTB2-sfGFP; WHI5-mCherry* | *W303; MATα; ADE2; HIS3; leu2Δ0; LYS2; ura3Δ0; trp1∆1; MET15; HTB2-sfGFP::kanMX4; WHI5-mCherry::SpHIS5* | This study |
| YCG60 | *S288C (BY4741)* | *HTB2-sfGFP; fkh1Δ* | *BY4741; MATa; ADE2; his3Δ1; leu2Δ0; ura3Δ0; TRP1; HTB2-sfGFP::kanMX4; fkh1::KanMX4* | This study |
| YCG64 | *S288C (BY4741)* | *HTB2-sfGFP; hcm1Δ* | *BY4741; MAT*α*; ADE2; his3Δ1; leu2Δ0; lys2Δ0; ura3Δ0; TRP1; HTB2-sfGFP::kanMX4; hcm1::KanMX4* | This study |
| YCG66 | *S288C (BY4741)* | *HTB2-sfGFP; dpb3Δ* | *BY4741; MAT*a*; ADE2; his3Δ1; leu2Δ0; lys2Δ0; ura3Δ0; TRP1; HTB2-sfGFP::kanMX4; dpbDPB3::KanMX4* | This study |
| YCG68 | *S288C (BY4741)* | *HTB2-sfGFP; ura7Δ* | *BY4741; MAT*α*; ADE2; his3Δ1; leu2Δ0; LYS2; ura3Δ0; TRP1; HTB2-sfGFP::kanMX4; ura7::KanMX4* | This study |
| YCG70 | *S288C (BY4741)* | *HTB2-sfGFP; sic1Δ* | *BY4741; MAT*α*; ADE2; his3Δ1; leu2Δ0; LYS2; ura3Δ0; TRP1; HTB2-sfGFP::kanMX4; sic1::KanMX4* | This study |
| YCG72 | *S288C (BY4741)* | *HTB2-sfGFP; cln1Δ* | *BY4741; MAT*a*; ADE2; his3Δ1; leu2Δ0; lys2Δ0; ura3Δ0; TRP1; HTB2-sfGFP::kanMX4; cln1::KanMX4* | This study |
| YCG75 | *S288C (BY4741)* | *HTB2-sfGFP; cln2Δ* | *BY4741; MAT*a*; ADE2; his3Δ1; leu2Δ0; LYS2; ura3Δ0; TRP1; HTB2-sfGFP::kanMX4; cln2::KanMX4* | This study |
| YCG77 | *S288C (BY4741)* | *HTB2-sfGFP; swi4Δ* | *BY4741; MAT*α*; ADE2; his3Δ1; leu2Δ0; LYS2; ura3Δ0; TRP1; HTB2-sfGFP::kanMX4; swi4::KanMX4* | This study |
| YCG79 | *S288C (BY4741)* | *HTB2-sfGFP; slk19Δ* | *BY4741; MAT*a*; ADE2; his3Δ1; leu2Δ0; LYS2; ura3Δ0; TRP1; HTB2-sfGFP::kanMX4; slk19::KanMX4* | This study |
| YCG85 | *S288C (BY4741)* | *HTB2-sfGFP; whi5Δ* | *BY4741; MAT*a*; ADE2; his3Δ1; leu2Δ0; ura3Δ0; TRP1; HTB2-sfGFP::kanMX4; whi5::KanMX4* | This study |
| YCG86 | *S288C (BY4741)* | *HTB2-sfGFP; whi5Δ* | *BY4741; MAT*a*; ADE2; his3Δ1; leu2Δ0; ura3Δ0; TRP1; HTB2-sfGFP::kanMX4; whi5::KanMX4* | This study |
| YCG87 | *S288C (BY4741)* | *HTB2-sfGFP; whi5Δ* | *BY4741; MAT*a*; ADE2; his3Δ1; leu2Δ0; ura3Δ0; TRP1; HTB2-sfGFP::kanMX4; whi5::KanMX4* | This study |
| YCG88 | *S288C (BY4741)* | *HTB2-sfGFP; whi5Δ* | *BY4741; MAT*a*; ADE2; his3Δ1; leu2Δ0; ura3Δ0; TRP1; HTB2-sfGFP::kanMX4; whi5::KanMX4* | This study |
| YCG89 | *S288C (BY4741)* | *HTB2-sfGFP; whi5Δ* | *BY4741; MAT*α*; ADE2; his3Δ1; leu2Δ0; ura3Δ0; TRP1; HTB2-sfGFP::kanMX4; whi5::KanMX4* | This study |
| YCG91 | *S288C (BY4741)* | *HTB2-sfGFP; fkh2Δ* | *BY4741; MAT*a*; ADE2; his3Δ1; leu2Δ0; ura3Δ0; TRP1; HTB2-sfGFP::kanMX4; fkh2::KanMX4* | This study |
| YCG93 | *S288C (BY4741)* | *HTB2-sfGFP; swe1Δ* | *BY4741; MAT*α*; ADE2; his3Δ1; leu2Δ0; ura3Δ0; TRP1; HTB2-sfGFP::kanMX4; swe1::KanMX4* | This study |
| YCG97 | *S288C (BY4741)* | *HTB2-sfGFP; mrc1Δ* | *BY4741; ADE2; his3Δ1; leu2Δ0; ura3Δ0; TRP1; HTB2-sfGFP::kanMX4; mrc1::KanMX4* | This study |
| YCG99 | *S288C (BY4741)* | *HTB2-sfGFP; rad27Δ* | *BY4741; MAT*α*; ADE2; his3Δ1; leu2Δ0; ura3Δ0; TRP1; HTB2-sfGFP::kanMX4; rad27::KanMX4* | This study |
| YCG103 | *S288C (BY4741)* | *HTB2-sfGFP; tda3Δ* | *BY4741; MAT*a*; ADE2; his3Δ1; leu2Δ0; ura3Δ0; TRP1; HTB2-sfGFP::kanMX4; tda3::KanMX4* | This study |
| YCG105 | *S288C (BY4741)* | *HTB2-sfGFP; clb5Δ* | *BY4741; ADE2; his3Δ1; leu2Δ0; ura3Δ0; TRP1; HTB2-sfGFP::kanMX4; clb5::KanMX4* | This study |
| YCG109 | *S288C (BY4741)* | *HTB2-sfGFP; cdh1Δ* | *BY4741; MAT*α*; ADE2; his3Δ1; leu2Δ0; met15Δ0; ura3Δ0; TRP1; HTB2-sfGFP::kanMX4; cdh1::KanMX4* | This study |
| YCG111 | *S288C (BY4741)* | *HTB2-sfGFP; dia2Δ* | *BY4741; MAT*α*; ADE2; his3Δ1; leu2Δ0; ura3Δ0; TRP1; HTB2-sfGFP::kanMX4; dia2::KanMX4* | This study |
| YCG115 | *S288C (BY4741)* | *HTB2-sfGFP; dbf2Δ* | *BY4741; MAT*a*; ADE2; his3Δ1; leu2Δ0; ura3Δ0; TRP1; HTB2-sfGFP::kanMX4; dbf2::KanMX4* | This study |
| YCG119 | *S288C (BY4741)* | *HTB2-sfGFP; cln3Δ* | *BY4741; MAT*a*; ADE2; his3Δ1; leu2Δ0; ura3Δ0; TRP1; HTB2-sfGFP::kanMX4; cln3::KanMX4* | This study |
| YCG121 | *S288C (BY4741)* | *HTB2-sfGFP; clb2Δ* | *BY4741; MAT*α*; ADE2; his3Δ1; leu2Δ0; met15Δ0; ura3Δ0; TRP1; HTB2-sfGFP::kanMX4; clb2::KanMX4* | This study |
| YCG127 | *S288C (BY4741)* | *HTB2-sfGFP; bck2Δ* | *BY4741; MAT*a*; ADE2; his3Δ1; leu2Δ0; MET15; ura3Δ0; TRP1; HTB2-sfGFP::kanMX4; bck2::KanMX4* | This study |
| YCG131 | *S288C (BY4741)* | *HTB2-sfGFP; clb5Δ; clb6Δ* | *BY4741; ADE2; his3Δ1; LEU2; ura3Δ0; TRP1; HTB2-sfGFP::kanMX4; clb5::KanMX4; clb6::LEU2* | This study |
| YCG132 | *S288C (BY4741)* | *HTB2-sfGFP; clb5Δ; clb6Δ* | *BY4741; ADE2; his3Δ1; LEU2; ura3Δ0; TRP1; HTB2-sfGFP::kanMX4; clb5::KanMX4; clb6::LEU2* | This study |
| YCG133 | *S288C (BY4741)* | *HTB2-sfGFP; clb5Δ; clb6Δ* | *BY4741; ADE2; his3Δ1; LEU2; ura3Δ0; TRP1; HTB2-sfGFP::kanMX4; clb5::KanMX4; clb6::LEU2* | This study |
| YCG134 | *S288C (BY4741)* | *HTB2-sfGFP; clb5Δ; clb6Δ* | *BY4741; ADE2; his3Δ1; LEU2; ura3Δ0; TRP1; HTB2-sfGFP::kanMX4; clb5::KanMX4; clb6::LEU2* | This study |
| YCG135 | *S288C (BY4741)* | *HTB2-sfGFP; clb5Δ; clb6Δ* | *BY4741; ADE2; his3Δ1; LEU2; ura3Δ0; TRP1; HTB2-sfGFP::kanMX4; clb5::KanMX4; clb6::LEU2* | This study |
| YCG136 | *S288C (BY4741)* | *HTB2-sfGFP; clb5Δ; clb6Δ* | *BY4741; ADE2; his3Δ1; LEU2; ura3Δ0; TRP1; HTB2-sfGFP::kanMX4; clb5::KanMX4; clb6::LEU2* | This study |
| YCG137 | *S288C (BY4741)* | *HTB2-sfGFP; clb5Δ; clb6Δ* | *BY4741; ADE2; his3Δ1; LEU2; ura3Δ0; TRP1; HTB2-sfGFP::kanMX4; clb5::KanMX4; clb6::LEU2* | This study |
| YCG138 | *S288C (BY4741)* | *HTB2-sfGFP; clb5Δ; clb6Δ* | *BY4741; ADE2; his3Δ1; LEU2; ura3Δ0; TRP1; HTB2-sfGFP::kanMX4; clb5::KanMX4; clb6::LEU2* | This study |
| YCG141 | *S288C (BY4741)* | *HTB2-sfGFP; clb5Δ; clb6Δ* | *BY4741; MATα; ADE2; his3Δ1; LEU2; ura3Δ0; TRP1; HTB2-sfGFP::kanMX4; clb5::KanMX4; clb6::LEU2* | This study |
| YCG142 | *S288C (BY4741)* | *HTB2-sfGFP; clb5Δ; clb6Δ* | *BY4741; MATα; ADE2; his3Δ1; LEU2; ura3Δ0; TRP1; HTB2-sfGFP::kanMX4; clb5::KanMX4; clb6::LEU2* | This study |
| YCG143 | *S288C (BY4741)* | *HTB2-sfGFP; clb5Δ; clb6Δ* | *BY4741; MATα; ADE2; his3Δ1; LEU2; ura3Δ0; TRP1; HTB2-sfGFP::kanMX4; clb5::KanMX4; clb6::LEU2* | This study |
| YCG144 | *S288C (BY4741)* | *HTB2-sfGFP; clb5Δ; clb6Δ* | *BY4741; MATα; ADE2; his3Δ1; LEU2; ura3Δ0; TRP1; HTB2-sfGFP::kanMX4; clb5::KanMX4; clb6::LEU2* | This study |

**Plasmid list**

| Name | *Background* | *Relevant Genotype* | *Genotype* | Origin |
| --- | --- | --- | --- | --- |
| pMaM4 | sfGFP cloned into pCM76 | sfGFP-kanMX | pFA6a-sfGFP-kanMX, AmpR | Knop M. |
