# Supplementary material for: Multiple inputs ensure yeast cell size homeostasis during cell cycle progression
Source: eLife. 2018 Jul 4;7:e34025. doi: 10.7554/eLife.34025 (PMC6085122; doi:10.7554/eLife.34025)
Supplement: Supplementary file 2. [file elife-34025-supp2.docx]

|  |  | Tdiv | SEM | p-val | TG1 | SEM | p-val | TS | SEM | p-val | TG2 | SEM | p-val | Tana | SEM | p-val |
| --- | --- | --- | --- | --- | --- | --- | --- | --- | --- | --- | --- | --- | --- | --- | --- | --- |
| WT | M | 76,5 | 0,1 | N/A | 19 | 0,1 | N/A | 29,5 | 0,1 | N/A | 15,8 | 0,1 | N/A | 12,2 | 0 | N/A |
| WT | D | 108,2 | 0,3 | N/A | 45,5 | 0,3 | N/A | 36,7 | 0,2 | N/A | 14,7 | 0,1 | N/A | 11,4 | 0,1 | N/A |
| fkh1 | M | 75,7 | 0,3 | N.S. | 19,6 | 0,3 | *** | 27,3 | 0,3 | *** | 14,9 | 0,2 | *** | 13,9 | 0,1 | *** |
| fkh1 | D | 105,5 | 0,9 | *** | 43,5 | 0,8 | *** | 35,3 | 0,6 | *** | 13,5 | 0,3 | *** | 13,3 | 0,2 | *** |
| hcm1 | M | 84,7 | 0,5 | *** | 18,6 | 0,3 | *** | 28,3 | 0,4 | *** | 22,6 | 0,4 | *** | 15,2 | 0,2 | *** |
| hcm1 | D | 103 | 1,1 | *** | 34,3 | 0,8 | *** | 33,7 | 0,7 | *** | 20 | 0,5 | *** | 15 | 0,2 | *** |
| dpb3 | M | 89,8 | 0,6 | *** | 20 | 0,4 | *** | 40,9 | 0,6 | *** | 17,3 | 0,4 | *** | 11,7 | 0,2 | ** |
| dpb3 | D | 109,5 | 0,9 | *** | 40,3 | 0,9 | *** | 43,9 | 0,8 | *** | 14 | 0,5 | *** | 11,2 | 0,2 | *** |
| ura7 | M | 87,1 | 0,6 | *** | 20,3 | 0,3 | *** | 34,2 | 0,5 | *** | 20,2 | 0,4 | *** | 12,5 | 0,2 | ** |
| ura7 | D | 107,5 | 1 | *** | 37,8 | 0,8 | *** | 39,8 | 0,8 | *** | 18 | 0,6 | *** | 11,9 | 0,2 | *** |
| sic1 | M | 83,4 | 0,6 | *** | 18,5 | 0,3 | N.S. | 31,8 | 0,4 | *** | 20,9 | 0,4 | *** | 12,3 | 0,1 | *** |
| sic1 | D | 100 | 1,3 | *** | 27 | 0,7 | *** | 34,2 | 0,7 | *** | 26 | 0,9 | *** | 12,7 | 0,2 | *** |
| cln1 | M | 78 | 0,4 | *** | 17,4 | 0,3 | *** | 29,9 | 0,4 | N.S. | 18 | 0,4 | *** | 12,8 | 0,2 | ** |
| cln1 | D | 103,5 | 1,1 | *** | 39,3 | 0,9 | *** | 35,6 | 0,8 | *** | 16,8 | 0,5 | ** | 11,7 | 0,2 | ** |
| cln2 | M | 94,8 | 0,7 | *** | 24,1 | 0,5 | *** | 35,7 | 0,5 | *** | 21,8 | 0,5 | *** | 13,2 | 0,2 | *** |
| cln2 | D | 120,6 | 1,1 | *** | 47,9 | 1 | *** | 40,2 | 0,8 | *** | 19,8 | 0,5 | *** | 12,5 | 0,2 | * |
| swi4 | M | 84,9 | 0,4 | *** | 20,3 | 0,3 | *** | 32,2 | 0,4 | *** | 19,7 | 0,3 | *** | 12,6 | 0,2 | ** |
| swi4 | D | 107 | 0,8 | *** | 36 | 0,7 | *** | 39,8 | 0,7 | *** | 19,5 | 0,4 | *** | 11,8 | 0,2 | *** |
| slk19 | M | 87,3 | 0,7 | *** | 20,7 | 0,6 | *** | 33 | 0,6 | *** | 16,3 | 0,5 | N.S. | 17,3 | 0,3 | *** |
| slk19 | D | 110,9 | 1,5 | *** | 41,8 | 1,2 | *** | 38 | 1 | *** | 15 | 0,6 | ** | 16,1 | 0,4 | *** |
| whi5 | M | 88,4 | 0,4 | *** | 22,3 | 0,3 | *** | 34,5 | 0,3 | *** | 18,4 | 0,3 | *** | 13,2 | 0,1 | *** |
| whi5 | D | 110,4 | 0,7 | *** | 39,6 | 0,6 | *** | 40,3 | 0,5 | *** | 18 | 0,4 | *** | 12,6 | 0,1 | *** |
| fhk2 | M | 85,3 | 0,7 | *** | 21,3 | 0,5 | *** | 32,5 | 0,6 | *** | 17,4 | 0,5 | ** | 14,1 | 0,2 | *** |
| fhk2 | D | 101,5 | 1,2 | *** | 36,2 | 1 | *** | 35,9 | 0,8 | *** | 16,2 | 0,6 | N.S. | 13,2 | 0,3 | *** |
| swe1 | M | 74,8 | 0,4 | *** | 19,1 | 0,3 | N.S. | 28,7 | 0,3 | N.S. | 14,5 | 0,3 | *** | 12,5 | 0,1 | N.S. |
| swe1 | D | 111,3 | 1,2 | *** | 48,9 | 1,1 | *** | 37,9 | 0,8 | *** | 13,3 | 0,4 | *** | 11,4 | 0,2 | *** |
| mrc1 | M | 94,3 | 0,7 | *** | 18,2 | 0,5 | *** | 32,4 | 0,6 | *** | 29,2 | 0,6 | *** | 14,5 | 0,2 | *** |
| mrc1 | D | 107,9 | 0,9 | *** | 31 | 0,7 | *** | 35,4 | 0,7 | *** | 27,6 | 0,6 | *** | 13,9 | 0,2 | *** |
| rad27 | M | 101 | 0,8 | *** | 21,2 | 0,4 | *** | 38,6 | 0,6 | *** | 27,8 | 0,6 | *** | 13,4 | 0,2 | *** |
| rad27 | D | 119,7 | 1,1 | *** | 38,8 | 0,8 | *** | 42,3 | 0,7 | *** | 25,9 | 0,7 | *** | 12,7 | 0,2 | *** |
| tda3 | M | 78,7 | 0,4 | *** | 19,3 | 0,3 | N.S. | 30,1 | 0,4 | N.S. | 16,4 | 0,4 | N.S. | 12,9 | 0,2 | *** |
| tda3 | D | 114,5 | 1,2 | *** | 48,3 | 1,2 | *** | 39,6 | 0,9 | *** | 14,8 | 0,5 | *** | 11,8 | 0,2 | * |
| clb5 | M | 88,8 | 0,8 | *** | 18 | 0,4 | *** | 36 | 0,6 | *** | 22,6 | 0,6 | *** | 12,3 | 0,2 | * |
| clb5 | D | 108 | 1,3 | *** | 37,7 | 1,1 | *** | 40,9 | 0,9 | *** | 18,3 | 0,8 | *** | 11,2 | 0,2 | *** |
| dia2 | M | 116,3 | 1,1 | *** | 18,9 | 0,4 | ** | 48,7 | 0,9 | *** | 34,4 | 0,8 | *** | 14,2 | 0,2 | *** |
| dia2 | D | 120,7 | 1,3 | *** | 30,9 | 0,8 | *** | 45,5 | 0,9 | *** | 31,4 | 0,8 | *** | 12,9 | 0,2 | *** |
| dbf2 | M | 89,5 | 0,7 | *** | 23,2 | 0,5 | *** | 32,9 | 0,6 | *** | 20 | 0,5 | *** | 13,3 | 0,2 | *** |
| dbf2 | D | 101,9 | 1 | *** | 34,5 | 0,9 | *** | 37 | 0,8 | *** | 18,1 | 0,6 | *** | 12,4 | 0,2 | N.S. |
| cln3 | M | 77,2 | 0,5 | * | 25,1 | 0,4 | *** | 27,8 | 0,4 | *** | 11,9 | 0,4 | *** | 12,4 | 0,2 | ** |
| cln3 | D | 98,3 | 1,1 | *** | 42,3 | 1 | *** | 31,9 | 0,7 | *** | 12,7 | 0,4 | *** | 11,5 | 0,2 | *** |
| clb2 | M | 90 | 0,9 | *** | 20,3 | 0,5 | ** | 32,3 | 0,7 | * | 22,1 | 0,7 | *** | 15,1 | 0,3 | *** |
| clb2 | D | 98,3 | 1,2 | *** | 26,3 | 0,8 | *** | 32,7 | 0,6 | *** | 24,6 | 0,7 | *** | 14,7 | 0,3 | *** |
| bck2 | M | 71,5 | 0,4 | *** | 18,7 | 0,4 | N.S. | 27,4 | 0,4 | ** | 13,1 | 0,3 | *** | 12,4 | 0,2 | N.S. |
| bck2 | D | 110,3 | 1,5 | *** | 47,6 | 1,4 | *** | 39 | 1,1 | *** | 12,2 | 0,4 | *** | 11,5 | 0,2 | *** |
| cdh1 | M | 80,2 | 1,2 | ** | 16,1 | 0,7 | *** | 30 | 0,9 | N.S. | 21,1 | 1,1 | *** | 13 | 0,4 | * |
| cdh1 | D | 97,3 | 2,6 | *** | 26,1 | 2 | *** | 36,9 | 1,8 | *** | 22,4 | 1,3 | *** | 11,9 | 0,3 | N.S. |

**Table 1**
